# Thrombotic Microangiopathy in IgA Nephropathy

**DOI:** 10.5812/ircmj.10234

**Published:** 2013-12-05

**Authors:** Hamid Nasri

**Affiliations:** 1 Department of Nephrology, Division of Nephropathology, Isfahan University of Medical Sciences, Isfahan, IR Iran

**Keywords:** Thrombosis, Thrombotic Microangiopathies, Nephropathy

**Dear Editor,**

Thrombotic microangiopathy (TMA) occurs in IgA nephropathy, but is uncommon in the setting of IgA nephropathy. Recently an article published by El Karoui and colleagues, entitled “A clinicopathologic study of thrombotic microangiopathy in IgA nephropathy” (1). They retrospectively examined a series of 128 patients diagnosed with IgA nephropathy (IgAN) between 2002 and 2008 who had a mean follow-up of 44 ± 27 months. They found that, 53 % presented with lesions of thrombotic microangiopathy (TMA), acute or organized, in arteries and / or arterioles. They concluded that, lesions of TMA are frequent in IgA nephropathy and may occur in normotensive patients with near-normal renal histology (1). However, we would like to remind a few points about TMA in IgA nephropathy. In a study on 102 primary IgA nephropathy patients, we found, morphologic lesions of TMA in 2 % of our patients, while all of them had malignant hypertension (2). In our study 71.6 % of patients were male. The mean age of the patients was 37.7 year. Morphologic variables of MEST classification was as follows; M1: 90.2 %, E: 32 %, S: 67 % also in grads I and II were in 30 % and 19 % respectively, while 51 % were in grade zero. Previously in a study on a small group of IgAN patients with thrombotic microangiopathy injury, Chang et al. (3) found, association of TMA with advanced stages and severe proteinuria of IgA nephropathy. Few publications existed regarding the TMA in IgA nephropathy. However, it is uncommon in the setting of IgA nephropathy and its significance, as a concomitant histologic finding, is unclear (4, 5). It is possible that IgAN has different presentation between different regions (6-8). However, the reports of overlapping morphologic lesions of TMA and IgAN are poorly understood and debatable and needs more attention in larger series of IgAN (8-10).
